# Counseling Tobacco Smoke Exposure Reduction Measures in Pediatrics: A Quality Improvement Project

**DOI:** 10.1097/pq9.0000000000000588

**Published:** 2022-09-15

**Authors:** Kevin Ferguson, Sankaran Krishnan, Emily Sullivan, Shyall Bhela, Allen J. Dozor, John J. Welter

**Affiliations:** From the * Maria Fareri Children’s Hospital at WMC Health, Valhalla, N.Y.; † Boston Children’s Health Physicians, Hawthorne, N.Y.; ‡ New York Medical College, Valhalla, New York; § College of the Holy Cross, Worcester, Ma.

## Abstract

With over 40% of children in the USA exposed to tobacco smoke, the AAP recommends tobacco smoke exposure (TSE) assessment during clinic visits. We aimed to increase the rates of TSE screening and provider counseling regarding TSE reduction using an evidence-based approach.

**Methods:**

We conducted the project at a large pediatric pulmonology practice. Baseline caregiver surveys and medical record review of TSE documentation took place in July/August, 2019. From September 2019 to July 2021, PDSA cycles were conducted to increase TSE screening and reduce counseling.

**Results:**

Before starting the project, 18% of smoking caregivers acknowledged smoking in the home and 41% in the car. While caregivers strongly desired to decrease TSE (median 9.4/10 on Likert scale), physician counseling of TSE reduction was offered only to 44%. PDSA cycles led to refining our patient passport, a document used during patient intake, which increased screening of TSE from 46% to 85%. Creating an educational handout in our electronic record addressing TSE increased TSE reduction counseling from 44% to 80% of children with smokers in the home.

**Conclusions:**

Incorporating TSE screening into established nursing documentation of vital signs led to the sustained screening of TSE among children in a pediatric pulmonology practice. Embedding educational material in our electronic record and changes in clinic processes increased TSE reduction counseling. Similar changes could improve rates of counseling caregivers of other guidelines aimed to improve the children’s health.

## INTRODUCTION

Forty percent of children in the US are exposed to environmental tobacco smoke, which substantially increases the risk for multiple morbidities.^1^ There are three types of tobacco smoke exposure (TSE). Firsthand smoke refers to smoke inhaled by a smoker.^2^ Secondhand smoke is produced by a tobacco product being smoked by one person and then inhaled by another.^2^ Thirdhand smoke contaminates surfaces exposing nonusers by either direct contact or inhalation.^2,3^ Childhood tobacco smoke exposure (TSE) increases the risks of sudden infant death, childhood obesity, behavior problems, neurocognitive deficits, wheezing, more severe asthma, more severe bronchiolitis, pneumonia, middle ear infections, chronic cough, and cancer.^2^ Because their bodies are small and developing, infants and young children are especially vulnerable to the toxins in secondhand smoke.^4^ Secondhand smoke exposure can slow lung growth and cause children with asthma to experience more frequent and severe attacks.^4^

Thirdhand smoke (THS) originates from the direct contamination of surfaces (eg, smokers’ bodies and clothes, indoor furnishings and surfaces, and building materials) with hazardous volatile organic compounds (VOCs) from tobacco combustion.^5^ THS exposure occurs via inhalation of evaporated gases, suspended dusts, or VOCs condensed into aerosols, along with ingestion or dermal exposure via surfaces or dust.^5^ THS presents health risks to children as they breathe in toxic chemicals when they crawl on floors, sit in cars, or are held by adults who smoke.^3^

The American Academy of Pediatrics recommends assessment of TSE in both homes and cars during clinic visits for all children. This is especially important for our patient population who present with diseases caused or exacerbated by TSE.^2^ The 2018 study, *Kids Safe and Smokefree (KiSS) Multilevel Intervention to Reduce Child Tobacco Smoke Exposure,* demonstrated that the TSE intervention Ask, Advise, and Refer (AAR) reduces TSE as measured by urine cotinine (a nicotine metabolite).^6^ One of the main conclusions of this study was that the AAR intervention decreases TSE among children of smoking parents.^6^ Despite the American Academy of Pediatrics recommendation above and evidence of the potential effectiveness of counseling for TSE reduction, rates of tobacco counseling during pediatric medical visits are only slowing increasing, rising from 4.1% in 1997–1999 to 11.1% in 2009–2011.^7^

Our goal was to create a system that consistently screened for TSE and provided counseling for TSE reduction.

## METHODS

Our specific aims were to increase rates of TSE screening among all children seen in our pediatric pulmonology outpatient clinic, from a baseline of 46% to above 80% and to increase the percent of patients with positive TSE screen who receive TSE counseling from 44% to above 80% within 12 months.

### Context

We conducted the project at the main outpatient pediatric pulmonary subspecialty office of Boston Children’s Health Physicians in Hawthorne, NY. At this location, we have 17 pediatric pulmonologists seeing between 200 and 300 patients per week. All pediatric patients (0–21 years of age) presenting to clinic visits starting in August, 2019 were included in this project. Before initiation of this project, our nursing staff would measure vital signs, perform spirometry, and reconcile medications and allergies. Physicians conducted screening for TSE in the course of the clinical visit.

### Preliminary Evaluation

Our primary improvement team consisted of 1 Pediatric Pulmonary Fellow, 2 Pediatric Pulmonologists, 1 clinic nurse, and 1 front office clerical staff. Dartmouth Clinical Microsystem methodology^8^ was used to evaluate systems, engage stakeholders in the project, and identify areas for potential change. To determine the scope of the baseline TSE in our patients and rates of physician TSE counseling, we surveyed parents/caregivers before and after physician visits before initiating any system changes. The lead team for this project utilized a modified validated questionnaire designed to capture TSE in the home(s) or car(s) of our patients, the rates of our physicians asking about TSE, advising reduction, and referring to smoking cessation resources as well as caregiver willingness to eliminate TSE for their child (Supplemental Digital Content 1, materials and methods, http://links.lww.com/PQ9/A398).^9^ In August 2019, 133 caregivers approached during clinic check-in responded yes to someone living in the child’s home smoking anywhere. Eighty of these caregivers agreed to complete the survey, which was verbally administered either in the office or later by phone. Second and thirdhand smoke exposure was reviewed with them, along with eliminating tobacco smoke from homes and cars. They were also given or sent via email information from nysmokefree.com to encourage parents or relatives to contact the smoking cessation site for help with tobacco smoke cessation.^10^ This information was made available in English and Spanish.^10^

### Project Development

**Fig. 1.**
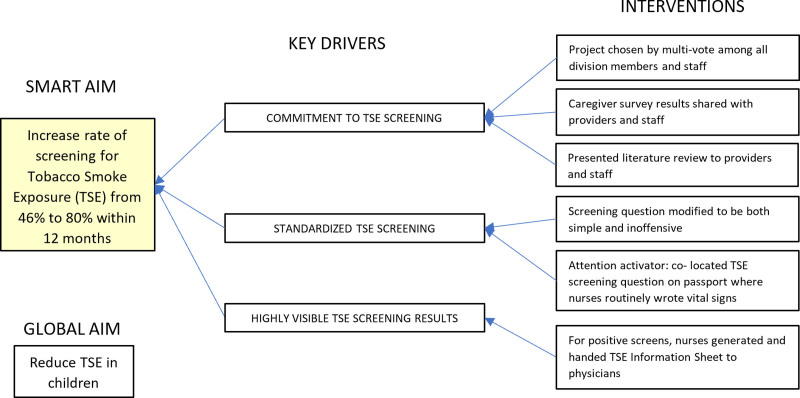
Key Driver Diagram for TSE screening.

**Fig. 2.**
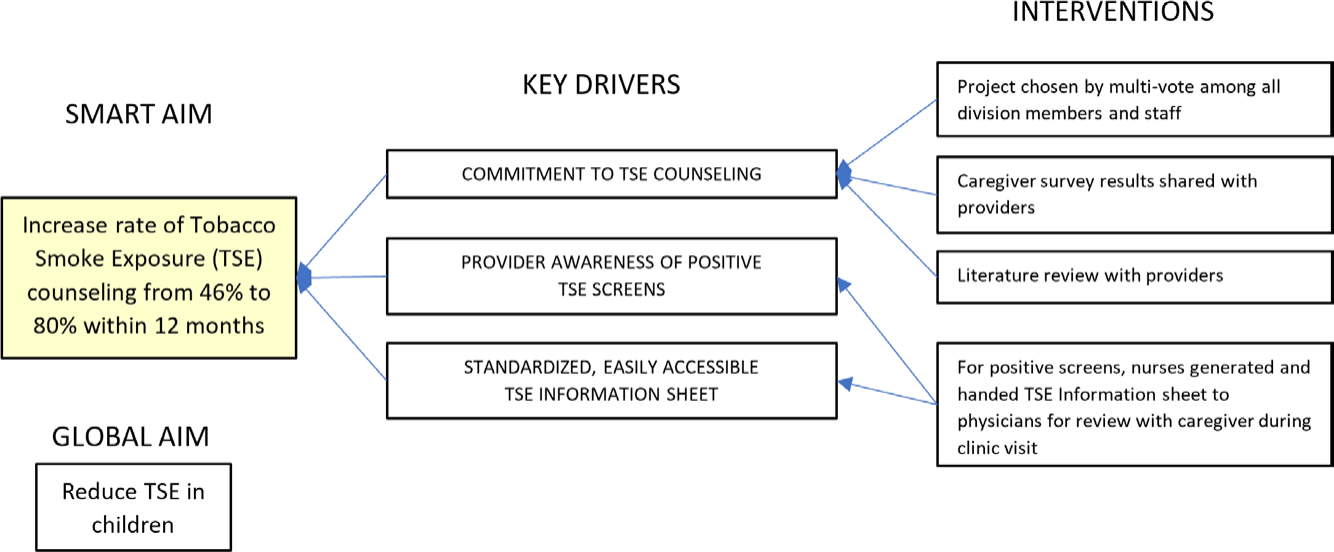
Key Driver Diagram for TSE counseling.

The lead team created Key Driver diagrams to identify issues with screening for, and counseling reduction of, TSE and design interventions (Figs. 1, 2).

To create ownership of the project amongst stakeholders, a literature review of TSE in pediatric patients and the results of our parent/caregiver surveys were presented to the entire division staff in August 2019. We polled physicians to choose which aspect of TSE in our patients they wanted to improve. Based on the positive impact of AAR in the KiSS study^6^ on urine cotinine levels in children, we chose to change processes and create tools that would draw physicians’ attention to TSE in their patients and create a tool for physicians to use during TSE reduction counseling.

### Interventions to Increase Screening and Counseling

Beginning in September, 2019 repeated changes aimed at increasing TSE screening were evaluated using plan-do-study-act cycles. Given the large number of providers in our practice, we sought to change workflow and leverage technology to improve outcomes with minimal impact on the time required to see patients. Throughout the project, the providers and staff shared data at weekly meetings.

### Role of Nursing

Our nurses played a key role in the process of screening for TSE. Previously, we had a very successful quality improvement project increasing rates of obtaining sputum cultures in people with Cystic Fibrosis by changing the collection of sputum samples from the physician to nursing during intake. Envisioning this success could be replicated, the first change tested in this project was having nursing staff begin screening for TSE in our patients during clinic intake. To capture as many opportunities as possible for TSE in a single question, we requested the nurses ask, “Is your child exposed to cigarettes or other tobacco smoke in anyone’s home or car?” Feedback from our nurses quickly informed us that this question offended some caregivers/parents. These caregivers/parents expressed that answering yes to the question meant they *allowed* their children to be exposed to tobacco smoke. The screening question was then simplified to “Does anyone in the home smoke any tobacco product?” With rare exceptions, this was an acceptable question to our families. Six weeks after introducing TSE screening into the nurses’ routines, we then asked our nurses to communicate this information on our “patient passport” to the physicians. The patient passport is a paper document utilized by our practice, which is generated upon clinic check-in and stays with the patient to facilitate communication between staff throughout the visit. The front office staff place the passport in a rack, which signals to the nurses the patient is ready for intake. Upon finishing intake, the nurses place the passport in a chart rack notifying the physician which patient is next to be seen. After the patient visit, the physician identifies the diagnoses and writes instructions for the front desk to facilitate scheduling follow-up appointments and referrals.

### Passport Tests of Change

At the onset of this project, we requested our nurses write on the passport “ETS+” (environmental tobacco smoke) when they encountered a caregiver with a positive TSE screen to notify the physician of TSE. After 4 weeks of writing “ETS+” on the passport, we reviewed our nurses’ progress and met with them to brainstorm how to improve their documentation on the passports. At their request, we revised our passport, adding “ETS Yes No” so that the nurse just had to circle the response. Six weeks later, we were still not at our goal of 80% screening for TSE. We noticed a universal nursing habit was to write the weight, height, and vital signs in a specific location on the paper passport. This led to the last change in our patient passport incorporating TSE documentation with vital signs recording (Supplemental Digital Content materials and methods 2, http://links.lww.com/PQ9/A398).

### TSE Reduction and Referral to Smoking Cessation Resources Handout Introduced

Concurrent with our attempts to increase screening for TSE, we created an information sheet presenting the risks posed by second and thirdhand smoke, containing non-judgmental statements to prompt discussion within families about creating smoke-free homes and cars, and provide resources for smokers to help with smoking cessation (Supplemental Digital Content materials and methods 3, http://links.lww.com/PQ9/A398). The information on this sheet was modified and drawn from several handouts from the American Academy of Pediatrics and California Department of Health websites.^3,11,12^ To improve ease of access, we placed the handout in our electronic health record document library, among other educational handouts distributed to parents/caregivers during clinic visits.

### Measures and Analysis

We defined screening as documentation of parent/caregiver questioning for smokers in the home on the patient’s passport at each clinic visit. We defined screening rates as the number of parent/caregivers screened for TSE as a proportion of patients seen in the clinic. All patient visits (including follow up of patients) were included in the measurement, and previous screening documentation did not count for the measurement. Screening for TSE was tracked from August 2019 through July 2021 and plotted on a control chart. Baseline screening rates were established by chart review from August 19^th^ through September 17^th^ 2019 before initiation of interventions. TSE reduction counseling was defined as documentation of the physician’s discussion of TSE reduction measures in the electronic health record. Rates of counseling were defined as the number of patients with documentation of TSE reduction counseling as a proportion of patients screening positive for TSE exposure. Patients counseled for TSE exposure within the prior month were not included in the measure. Data were analyzed using statistical process control charts with center line shifts determined using standard definitions of special cause variation.^13^

### Ethical Considerations

The New York Medical College Institutional Review Board deemed this project QI and exempted it from review. Confidentiality of patients, families, and providers was guarded in accordance with HIPAA guidelines.

## RESULTS

**Fig. 3.**
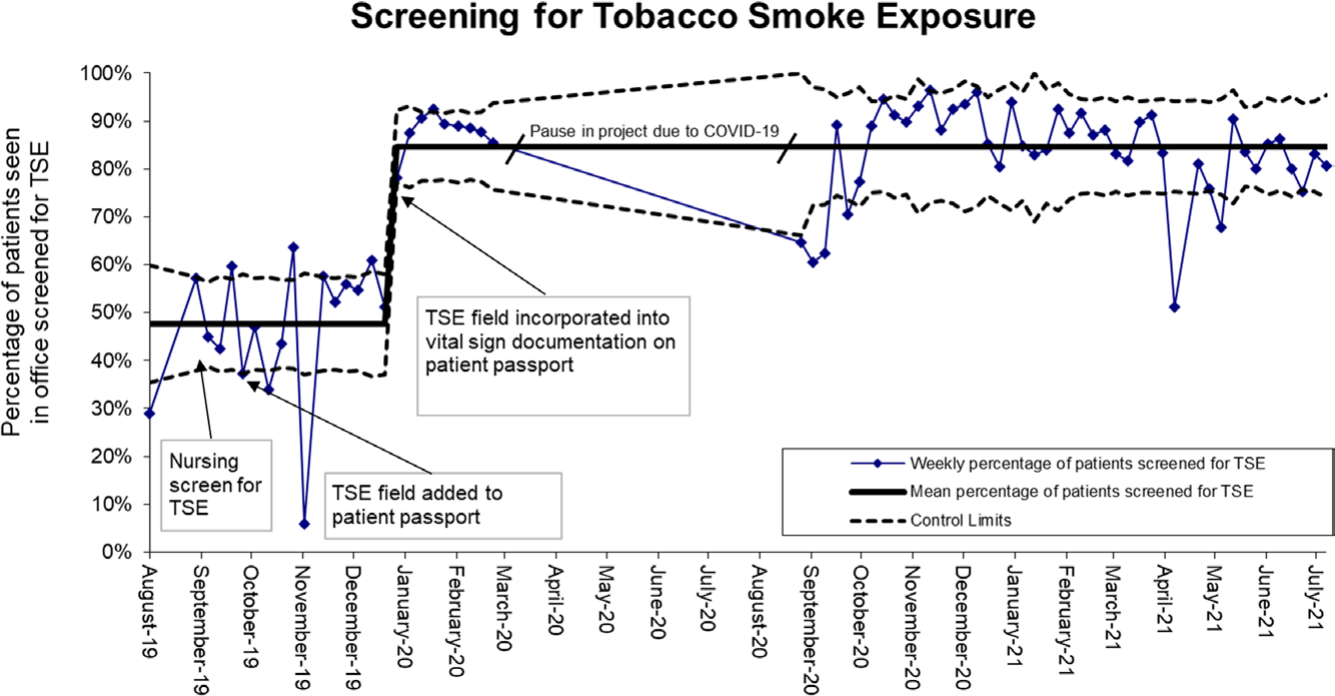
The percentage of patients seen in our office screened for tobacco smoke exposure. The numerator is the number of patients with documentation of tobacco smoke exposure (yes/no), and the denominator is the number of patients seen in clinic. Upper and lower limits represent 3σ.

All patients aged 0 to 21 years, a total of 10,444 clinic visits, from August 2019 through March 2020 and September 2020 through July 2021, were included in this project. The gap in data occurred during the sudden increase in virtual clinic visits and drop-off of patients being seen in person due to the onset of the COVID-19 pandemic. The project was placed on hold in April 2020 and resumed in September 2020 once in-person clinic visits rose above 80% of total visits. To obtain baseline data, in August 2019 80 of 133 caregivers of children with smokers in the home, agreed to be surveyed after a clinic visit detailing TSE, their desire to reduce TSE for their child and whether TSE reduction was discussed during the clinic visitme (Supplemental Digital Content materials and methods 1, http://links.lww.com/PQ9/A398). Among this group, 18% acknowledged smoking in the home and 41% in the car. Caregivers with smokers in the home had a high desire to decrease TSE (9.4/10 on a Likert scale), yet the survey revealed physician counseling of TSE reduction was only offered to 44% of caregivers with smokers in the home. Reviewing 387 patient charts, documentation of baseline screening for TSE occurred in 46%. TSE screening rates were charted overtime on a control chart (Fig. 3). The first test of change, having nurses screen for TSE during clinic intake, did not significantly improve TSE screening rates. The subsequent tests of change consisted of moving documentation to the patient’s passport and embedding the TSE screening exposure question into the passport, also did not lead to significant improvement in TSE screening. However, the last change in the patient passport incorporating TSE documentation with recording of height, weight, and vital signs led to an almost immediate and sustained increase in TSE screening rate to 85%, above our target of 80% set at the start of the project (Fig. 3).

**Fig. 4.**
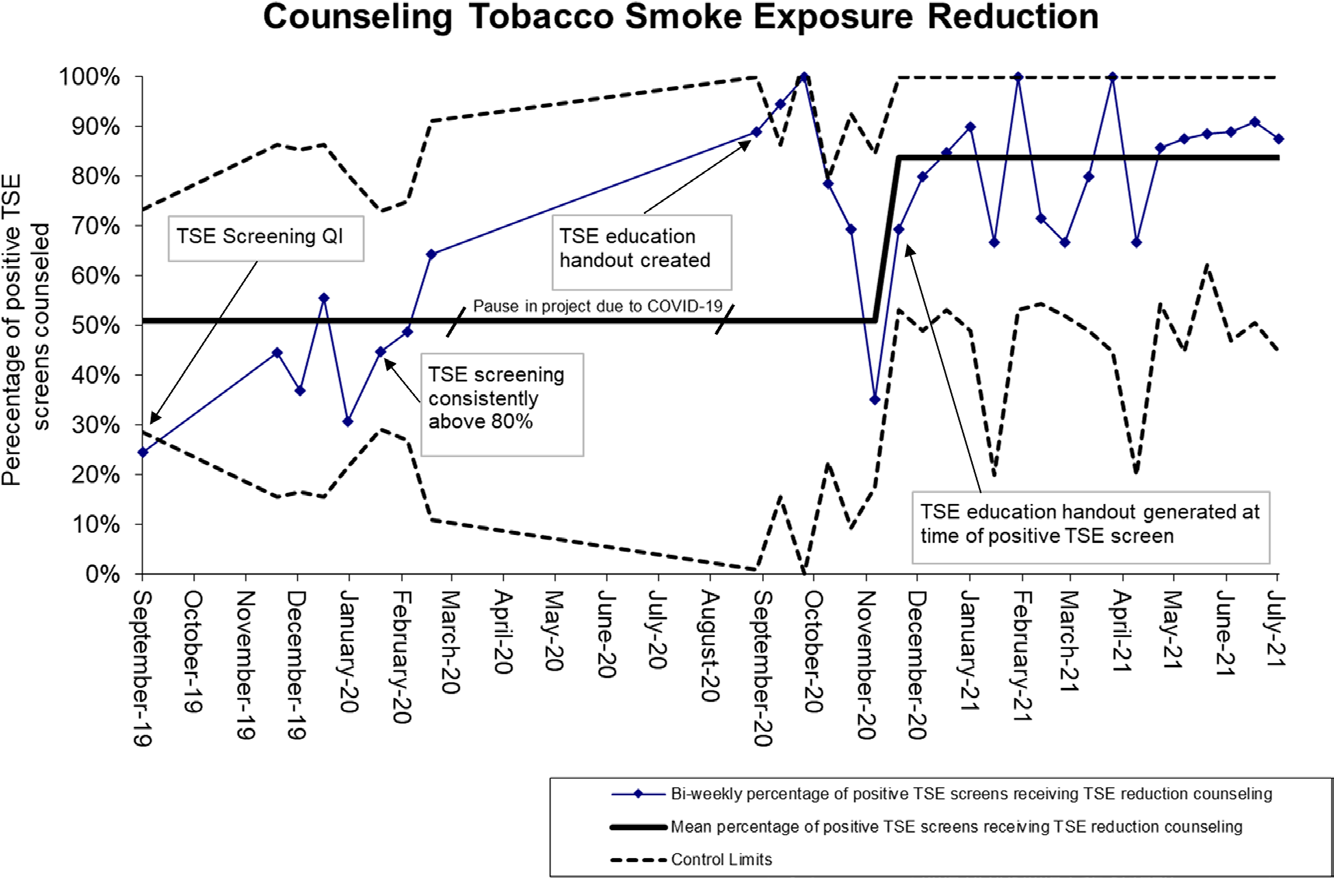
The percentage of patients screening positive for tobacco smokers in the home with counseling of tobacco smoke exposure reduction. The numerator is the number of patients with documentation of tobacco smoke exposure avoidance counseling and the denominator is the number of patients screening positive for tobacco smoke exposure. Upper and lower limits represent 3σ.

In September 2020, after sustained screening for TSE was achieved and in-person clinic visits rebounded after the start of the COVID-19 pandemic, we introduced our information sheet entitled “Let’s talk about…..Keeping Smoking out of our Homes and Cars” (Supplemental Digital Content materials and methods 3, http://links.lww.com/PQ9/A398). This paper handout aimed to eliminate second and thirdhand tobacco smoke from the environment and refer caregivers to smoking cessation resources. We also wanted this to be an easy tool that physicians could use, especially if they felt the time required for this conversation was a deterrent or if they were searching for succinct words to address this topic. This tool was printed out from the electronic health record and handed to caregivers by physicians when they identified patients with TSE. With initial education of our providers rates of counseling TSE avoidance rose above 80%; however, this quickly reverted to the baseline over the next 3 months. In December 2020, the nurses were tasked with generating and handing the TSE reduction handout to the patient’s physician for review with the caregivers during the clinic visit. This change led to a sustained increase in TSE reduction counseling in caregivers of our patients, from 44% to 80% (Fig. 4).

## DISCUSSION

TSE is a common and critical risk factor that adversely affects the respiratory health of infants, children, teenagers, and their parents. Stakeholder engagement for improving TSE screening and reduction counseling was high from the start of this project. Our practice holds quarterly quality improvement meetings, and new project ideas arise from brainstorming sessions at these meetings. All staff (clerks, nurses, and physicians) vote on new projects to create stakeholder ownership. Physicians expressed concern when shown data that they were missing TSE among their patients and thus not addressing this environmental exposure that can negatively impact patients. Nurses at our clinic expressed increased job satisfaction when they are involved in projects that can positively impact our patients.

Nursing feedback played a critical role in the success of this project. The initial TSE screening question was perceived as offensive by some caregivers/parents and thus impeded nursing willingness to screen for TSE. Our nurses revised the screening question to be more acceptable to our caregivers/parents, removing an initial barrier to TSE screening in our clinic.

Through nursing and physician workflow changes, we created a reliable system to identify and counsel TSE reduction in children. The most impressive and immediate change in results of our project came when we studied how our nurses utilize a tool already well established in our clinic, the paper patient passport. This system is simple, easy to use, and a highly effective method of communication in our clinic. Human factor engineering recognizes that quality improvement outcomes are not only reliant on system design, but they also are impacted by mental, physical, social, and behavioral activities carried out by health care professionals.^14^ Nurses working in a busy clinic with limited time per patient necessitated designing a tool that integrated into current work habits. To add TSE screening to their existing workflow, an attention activator was necessary. This was achieved by creation of a template on the passport located in the area where the nurses handwrote height, weight and vital signs (Supplemental Digital Content materials and methods 2, http://links.lww.com/PQ9/A398). By co-locating the screening question in this area, screening became incorporated with the vital signs documentation increasing our screening rate of TSE from 46% to 85%.

Embedding the TSE education into our electronic record allowed nurses to quickly print out the education sheets and give them to the patient’s physician to review with caregivers without impacting the time needed for patient intake. Physicians in our practice thought the tool was very useful in starting conversations regarding reducing TSE. Quite often, the person smoking in the home is not present for the clinic visit, and the tool gave the caregiver concrete suggestions to bring home to the smoker in the home. We believe this change was key to the increased TSE avoidance counseling rate amongst exposed children (Fig. 4).

Our project addresses an important recommendation from the American Academy of Pediatrics policy statement to protect children from tobacco, nicotine, and tobacco smoke by identifying less than ideal screening rates of TSE and increasing rates of screening and TSE reduction counseling.^2^ A potential weakness of our project was the accumulation of baseline data for just 1 month. Baseline screening and counseling rates were similar to our preliminary evaluation, performed before introduction of the project to the entire division, suggesting that they accurately reflected performance of our division’s TSE screening and counseling at that time. In addition, our global aim relies on the effectiveness of pediatrician counseling TSE reduction on decreasing children’s TSE. A meta-analysis of various studies focused on reducing children’s TSE did not identify a definitive means to reduce TSE.^15^ The KiSS study holds promise that screening and education impacts TSE in children, but further confirmation is required given the variability of TSE interventional studies to date.^6^ There are some limits to the generalizability of this project. We utilized a unique tool already in use at our clinic: the paper patient passport, which most likely is not present in clinics utilizing electronic health records. An electronic alternative would be to add a TSE field in the electronic record in an area where nursing is already entering patient information potentially increasing the likelihood of TSE screening. A key to the success of this project was generating a customized form in our electronic health record that some pediatric clinics may be unable to replicate. The cost and time required to track TSE screening and counseling data may impose barriers in some clinics. For our project, it took approximately 30 minutes per week of staff time to record screening and counseling data. We did not need additional staffing and utilized a hard copy paper tool, which could be easily created at any clinic, and can be used even by those with limited flexibility within their electronic records.

## CONCLUSIONS

TSE poses significant health risks to children exposed to it. Identifying children exposed to TSE is a critical step in creating opportunities for clinicians to intervene and counsel measures to reduce exposure. Next steps for our center are to identify more resources in our area to assist willing caregivers with tobacco smoke cessation. Through alterations to our clinic tools and workflow, which we believe can be replicated at other pediatric practices, we increased adherence to policies aimed at protecting children from tobacco smoke. Further, this type of intervention can be applied to a wide variety of modifiable risk screenings performed in pediatric clinics.

## ACKNOWLEDGMENT

Assistance with the study: Armando Ramirez assisted with data collection throughout the study.

## DISCLOSURE

The authors have no financial interest to declare in relation to the content of this article.

## Supplementary Material



## References

[1] Homa DM Neff LJ King BA.; Centers for Disease Control and Prevention (CDC). Vital signs: disparities in nonsmokers’ exposure to secondhand smoke–United States, 1999-2012. MMWR Morb Mortal Wkly Rep. 2015;64:103–108. 25654612 PMC4584848

[2] Farber HJ Groner J Walley S.; Section on Tobacco Control. Protecting Children From Tobacco, Nicotine, and Tobacco Smoke. Pediatrics. 2015;136:e1439–e1467. 26504135 10.1542/peds.2015-3110

[3] California Department of Public Health, California Tobacco Control Program. 2017. Thirdhand Smoke in Multi-unit Housing. https://www.cdph.ca.gov/Programs/CCDPHP/DCDIC/CTCB/CDPH%20Document%20Library/Community/EducationalMaterials/ThirdhandSmokeInMulti-unitHousingFactSheet.pdf.

[4] U.S. Dept. of Health and Human Services. The Health Consequences of Involuntary Exposure to Tobacco Smoke: A Report of the Surgeon General. Atlanta, Ga.: U.S. Dept. of Health and Human Services; 2006:xvii, 709p.

[5] Sheu R Stönner C Ditto JC. Human transport of thirdhand tobacco smoke: A prominent source of hazardous air pollutants into indoor nonsmoking environments. Sci Adv. 2020;6:eaay4109. 32181345 10.1126/sciadv.aay4109 PMC7056301

[6] Lepore SJ Collins BN Coffman DL. Kids Safe and Smokefree (KiSS) multilevel intervention to reduce child tobacco smoke exposure: long-term results of a randomized controlled trial. Int J Environ Res Public Health. 2018;15:E1239. 29895740 10.3390/ijerph15061239 PMC6025102

[7] Cawkwell PB Lee L Shearston J. The difference a decade makes: smoking cessation counseling and screening at pediatric visits. Nicotine Tob Res. 2016;18:2100–2105. 27613894 10.1093/ntr/ntw146 PMC5055743

[8] Nelson EC Batalden PB Godfrey MM. Part two: activating the organization and the dartmouth microsystem improvement curriculum. In: Nelson EC Batalden PB Godfrey MM, eds. Quality by Design: A Clinical Microsystems Approach, 1st ed. Jossey-Bass; 2007:199–270.

[9] Groner JA Hoshaw-Woodard S Koren G. Screening for children’s exposure to environmental tobacco smoke in a pediatric primary care setting. Arch Pediatr Adolesc Med. 2005;159:450–455. 15867119 10.1001/archpedi.159.5.450

[10] Roswell Park Cessation Services. 2019. Learning to quit – a simple guide to quit smoking [PDF]. https://www.nysmokefree.com/Downloads/Literature/LearningtoQuit_English.pdf.

[11] AAP Julius B. Richmond Center of Excellence. 2000. Smokefree homes and child health [PDF]. https://downloads.aap.org/AAP/PDF/SmokefreeCarsFactsheet.pdf.

[12] AAP Julius B. Richmond Center of Excellence. 2000. Smokefree Homes and Child Health [PDF]. https://downloads.aap.org/AAP/PDF/SmokefreeHomesFactsheet.pdf.

[13] Amin SG. Control charts 101: a guide to health care applications. Qual Manag Health Care. 2001;9:1–27. 10.1097/00019514-200109030-00003 11372500

[14] Karsh BT Holden RJ Alper SJ. A human factors engineering paradigm for patient safety: designing to support the performance of the healthcare professional. Qual Saf Health Care. 2006;15 Suppl 1:i59–i65. 17142611 10.1136/qshc.2005.015974 PMC2464866

[15] Behbod B Sharma M Baxi R. Family and career smoking control programmes for reducing children’s exposure to environmental tobacco smoke. Cochrane Database Syst Rev. 2018;1:CD001746. 29383710 10.1002/14651858.CD001746.pub4 PMC6491082

