# Supplementary material for: Counseling Tobacco Smoke Exposure Reduction Measures in Pediatrics: A Quality Improvement Project
Source: Pediatr Qual Saf. 2022 Sep 15;7(5):e588. doi: 10.1097/pq9.0000000000000588 (PMC9478329; doi:10.1097/pq9.0000000000000588)
Supplement: Supplementary file 1 [file pqs-7-e588-s001.pdf]

Screening Survey: Tobacco Smoke Exposure

Patient Name ________________ DOB_____________ Date of visit ______________

Primary Care Giver (mother, father, other) __________________________

Person completing survey ☐ Primary care giver ☐ Other _______________

Hi, my name is ________. We are performing a quality improvement effort in an attempt to reduce environmental tobacco smoke exposure for our patients. Would you be willing to take a very brief (1 to 2 minute) survey before you leave the office?

1. Does anyone who lives in the home smoke anywhere?
   ☐ Yes ☐ No (Thank them for their participation and end questionnaire)
2. Does the (primary caregiver from above) smoke?
   ☐ Yes ☐ No
3. Do others in the home smoke?
   ☐ Yes ☐ No (skip to question number 5)
4. If yes to question number 3: Do the others that smoke ever smoke inside the home?
   ☐ Yes ☐ No
5. Is your child ever in a car when people are smoking?
   ☐ Yes ☐ No
6. Is your child ever in a car that people have smoked in even when the child is not present?
   ☐ Yes ☐ No
7. During the doctor visit today, did you discuss ways to reduce tobacco smoke exposure for your child such as setting rules for smoking in the home or car, or tools to stop smoking such as medications or referral to resources like the NY State quitline?
   ☐ Yes ☐ No
8. On a scale from 1 to 10, how important is it to you to reduce tobacco smoke exposure to your child?

   Not at all 1 2 3 4 5 6 7 8 9 10 Extremely important

<u>Willingness to Quit and Eliminate Smoke Exposure</u>

How would you answer the following questions <u>after</u> today's office visit?

1. I would be willing to make a plan to quit smoking.
   Least Likely 1 2 3 4 5 Most likely
2. I would be willing to contact a referral source such as the NY state quitline.
   Least Likely 1 2 3 4 5 Most likely
3. I would be willing to cut down my number of cigarettes before quitting.
   Least Likely 1 2 3 4 5 Most likely
4. I know steps I need to take to eliminate smoke exposure to my child.
   Least Likely 1 2 3 4 5 Most likely
5. I have or will put in place bans on smoking in the car(s) and home.
   Least Likely 1 2 3 4 5 Most likely

Thank you for participating in this survey. Would you like this NY State quit line flyer to learn more about tools to help you stop smoking?

SDS, Materials and methods 1.

Pediatric Pulmonology, Allergy and Sleep Medicine
Boston Children's Health Physicians
Until every child is well™
19 Bradhurst Avenue Hawthorne NY, 10532-2140
Phone:(914)493-7585 Fax:(914)594-2350

Patient:
Date of Birth:
Encounter Date:
Historian:
Visit Type:

Patient Detail List

A	Allergies	N	Hypoventilation
B	Allergy Immunotherapy	O	Immunodeficiency
C	Asthma	P	Interstitial Lung Disease
D	Bronchopulmonary Dysplasia	Q	Narcolepsy
E	Bronchiectasis, Non-CF	R	Neuromuscular Disorder
F	Cough	S	Overweight
G	Cystic Fibrosis	T	Primary Ciliary Dyskinesia
H	CFRD	U	Sinusitis, recurrent
I	CRMS	V	Sleep Disorder, any
J	Environment Health Concern	W	Tracheostomy
K	Food Allergies	X	Underweight
L	Home Mechanical Ventilatory Support	Y	Urticaria
M	Home Oxygen Therapy	Z	COPD

ETS **( ) Yes**
( ) No

**WEIGHT**	
**HEIGHT**	
**PULSE**	
**O2 SAT**	

No follow-up necessary ( ) Scheduled at check-out ( )

Nutrition Follow-up:
RT Follow-up:
Social Work Follow-up:
Nurse Follow-up:

Next Physician Visit: **months**

SDS, Materials and methods 2.

Pediatric Pulmonology, Allergy and Sleep Medicine
Boston Children's Health Physicians
Until every child is well™
19 Bradhurst Avenue Hawthorne NY, 10532-2140
Phone:(914)493-7585 Fax:(914)594-2350

Let's talk about…..**Keeping Smoking out of our Homes and Cars**

Why? Because exposure to environmental tobacco smoke presents:

- Risk to Children: increased risk for **sudden infant death, ear infections, asthma, pneumonia and possibly cancer**
- Risk to Adults: increased risk of breathing problems, lung cancer, heart attacks and strokes

The facts…..

- ☐ **Secondhand smoke** is the smoke we breathe in when someone around us is smoking.
- ☐ **Thirdhand smoke** is tobacco smoke chemicals that are left behind when someone smokes in a home or car. The smoke does not simply blow away. Instead it sticks to surfaces such as walls, furniture and floors as well as to a person's hair, skin and clothing.
- ☐ Our babies and kids can be exposed to these chemicals when they crawl or play on the floor.
- ☐ Babies may eat these chemicals when they put toys or other objects in their mouths.
- ☐ Children's growing bodies make them more at risk for harm from these chemicals than adults.
- ☐ There is no safe level of tobacco smoke exposure

How to negotiate a **100% Smoke-Free Rule for your home and car**:

- ☑ Find a time with no distractions to talk calmly about setting No Smoking Rules.
- ☑ Agree on the common goal of protecting the health of your children and family.
- ☑ Use "I" statements, and give credit for the things the smoker is already doing:

"I know you already work hard not to smoke when the kids are around, but I think we can do more to protect them."

"I would like to have a rule about not allowing any smoking at any time in our home/car."

"I know it is hard to make changes and I really appreciate you trying."

"I would really like your help in asking our families/friends not to smoke in our home/car"

Additional Resources:

- Call NYS smokers quit line 1-866-NY-QUITS (1-866-697-8487) or visit https://www.nysmokefree.com
